# Cellular progression of neoplasia in the subcutis of mice after implantation of 3,4-benzpyrene.

**DOI:** 10.1038/bjc.1979.130

**Published:** 1979-06

**Authors:** F. R. Westwood, E. Longstaff, W. H. Butler

## Abstract

**Images:**


*Br. J. Cancer* (1979) **39**, 761

### CELLULAR PROGRESSION OF NEOPLASIA IN THE SUBCUTIS OF MICE AFTER IMPLANTATION OF 3,4-BENZPYRENE

F. R. WESTWOOD,* E. LONGSTAFF* AND W. H. BUTLER†

*From the *Central Toxicology Laboratories, and the †Pathology Section, Safety of Medicines Department, Imperial Chemical Industries Limited, Alderley Park, Macclesfield, Cheshire*

Received 1 November 1978 Accepted 17 February 1979

**Summary.—An implantation model has been used to investigate the cellular progression of chemically induced subcutaneous neoplasia in the mouse. Implantation of 3,4-benzpyrene induced persistent changes in the normal process of connective tissue formation around the implant. Light-microscope and autoradiographic studies have shown a temporal progression from aberrant filter- or muscle-associated cells through proliferative foci to large invasive sarcoma. Electron microscopy revealed that presarcomatous cell foci consisted of one of two different cell types. These were either spindle cells with ultrastructural characteristics similar to foreign-body-induced sarcoma, or cells with the ultrastructural features of rhabdomyosarcoma. The subsequent appearance of two histological groups of sarcoma that were ultrastructurally similar to the cells of the early proliferative foci indicated that both elements may progress to form tumours. However, the constituent cells of both groups of tumours displayed a broad histological and ultrastructural spectrum and the marked similarity between the undifferentiated cells of each suggested that both may have arisen from diverse differentiation of a common pluripotential cell such as the pericyte.**

ALTHOUGH repeated s.c. injection in rodents has been found to be of limited value for assessing the carcinogenicity of substances (Grasso & Golberg, 1966), s.c. implantation has been used with some success to trace the course of chemically induced neoplasia in the subcutis. Vasiliev (1959) and Vasiliev *et al.* (1962) produced a comprehensive treatise on the cell and tissue changes that occur after the s.c. implantation of polycyclic aromatic hydrocarbon (PAH) carcinogens in rats. These carcinogens produced changes in the cell and tissue reactions around the implanted material, consisting of inhibition of fibroblast differentiation and growth accompanied by leucocyte infiltration. This led to a profound distortion of normal fibrogenesis, and encapsulation of the implanted material. Similar responses were seen by Hooson *et al.* (1971, 1973) after s.c. injection of water-soluble carcinogens. Foci considered to be presarcomatous arose from cells remaining in the collagenized areas adjacent to the implanted carcinogen (Vasiliev *et al.*, 1962). These early foci were similar to those which arose after s.c. injection of a variety of carcinogenic substances (Carter, 1969, 1970) and were reported to consist of abnormal fibroblasts and fragmented distorted collagen and reticulin. These fibroblasts were pleomorphic, and mitotic figures were frequent. Although the cellular progression of induced s.c. neoplasia has been extensively examined, the cell types involved have not been conclusively established.

It was the aim of this work to locate and characterize the cell types involved in the progression of s.c. neoplasia induced by the carcinogen 3,4-benzpyrene (BP) using histology, autoradiography and electron microscopy. The implantation model developed by Lavelle (1973) and modified by us to assess carcinogenicity (Purchase *et al.*, 1976, 1978) involved s.c. implantation of Millipore filters overlaid with gelatin containing test substances. Using this technique it was found that tumours arose at the implant site in 100% of mice treated with BP within a 4-month period. This technique was therefore seen as an ideal model for the study of the cellular progression of neoplasia in the subcutis.

### METHODS

*Preparation of filter-disc implants.*—Millipore filters Type GSW 3000 (Millipore Corporation) with a pore size of 0·22 μm and 13 mm diameter were used. Test substances (5 mg BP or 4 mg pyrene) were suspended in 1 ml of dimethylsulphoxide. The suspensions were mixed with 9 ml of molten (50°C) aqueous 16% (w/v) gelatin (Type IIIA, Sigma Ltd).

The molten-gelatin mixtures (0·2ml aliquots) were dispensed on to the filters and left to gel at room temperature in sterile Petri dishes.

*Implantation and tissue sampling.*—Female Alderley Park strain Swiss albino mice 6–8 weeks old (weight range 20–40 g) were anaesthetized by i.p. injection of sodium pentobarbitone (Nembutal). An implant was inserted into the subcutis through a small incision made in the clipped skin in the lumbar area. The incision was closed with sutures. At various intervals after implantation the mice were killed by cervical dislocation. The area around the filter was sewn at 4 points to the underlying muscle tissue. This allowed the implant site to be excised without disturbing the spacial arrangement of the tissues.

Three groups of mice were implanted respectively with:

1. Gelatin control filters (66 mice).
2. Filters supporting 4 mg pyrene (0·1 mol equivalent) (31 mice).
3. Filters supporting 5 mg BP (0·1 mol equivalent) (145 mice).

Samples of implant-site tissue were taken for autoradiography, electron microscopy and histology 1, 2, 3 days and 1, 2, 4, 8, 9, 10, 11, 12, 13, 14 and 15 weeks after implantation.

*Histology.*—Implant-site tissues were fixed in Bouin's solution, cut into 5 pieces and embedded in paraffin wax. Sections (5 μm) were cut from each block and stained with haematoxylin and eosin, and with special stains where appropriate. Seventeen animals from each group were kept alive for up to 18 months or until tumours developed.

*Autoradiography.*—Forty-five minutes before being killed animals were injected i.p. with 1 μCi/g body weight $^3$H-thymidine (6-$^3$H, Radiochemical Centre, Amersham). Implant and adjacent tissues were excised and fixed in neutral buffered formal saline. Sections from these tissues were dipped in 50% aqueous 1% glycerol photographic nuclear emulsion (Type K2, Ilford) at 40°C and left at room temperature to dry for 3–4 h. Slides were desiccated (silica gel) at 4°C in light-tight boxes for 4 weeks before being developed in a 50% aqueous solution of D19 developer (Kodak). Slides were stained in Harris's haematoxylin solution.

*Electron microscopy.*—The implant and implant-site tissues were excised and fixed in 3% glutaraldehyde at 4°C. Tissue samples were removed after 12 h, and 1mm strips cut from the edge and middle of each. The material was post-fixed in Millonig's 1% osmium tetroxide, rinsed, dehydrated in graded alcohols and embedded in Araldite (Ciba-Geigy Ltd). Semi-thin sections (1 μm) were cut and stained with toluidine blue for light microscopy. Ultra-thin sections (70–90 nm) of areas of tissue of special interest were prepared for electron microscopy. The latter were stained with uranyl acetate and lead citrate and examined in an AEI EM6B microscope.

### RESULTS

### *Controls*

Inflammation appeared in the subpannicular connective tissue localized around the Millipore filter one day after implantation. Lymphocytes and polymorphs were present in large numbers in a proteinaceous exudate. Macrophages and fibroblasts were abundant by the 3rd day. Many fibroblast-like cells were in the S phase of cell division as detected by autoradiography. Cells incorporating $^3$H-TdR were also around the filter by the 7th day. After 2 weeks (Fig. 1) light and electron

FIG. 1.—Control tissue reaction 2 weeks after the implantation of a Millipore filter. The surface of the filter (MF) is coated by a condensed layer of macrophages (Mp) surrounded by fibroblasts (Fb) and collagen (Co). D, dermis; P.C., panniculus carnosus; C, capsule. ×300.

microscopy revealed that the capsule consisted of 1–3 layers of surface-attached macrophages that were in intimate contact and had extended long processes into the surfaces of the filter. Layers of orientated fibroblasts surrounded the macrophages, and were themselves surrounded by bundles of collagen. The capsule surrounding the filter resorbed over the following year of implantation, leaving the implant surface coated by a layer of foreign-body giant cells (FBG). The subpannicular connective tissue surrounded this layer. No s.c. tumours were discovered by palpation in any of the 14 mice implanted with control implants over an 18-month period.

*Pyrene*

Pyrene (4 mg/mouse) also induced an initial exudation and inflammation around the locality of the implant. Encapsulation by fibroblasts, macrophages and collagen occurred over the same period as with control implants. Changes different from gelatin controls were noted after 2 weeks, when irregular thick fibrous areas were seen within the normal capsule. The fibroblasts within these areas were associated with large amounts of collagen. Crystalline material, presumably pyrene, was present within these areas. The lesion nevertheless resolved, as described for the gelatin control. No s.c. tumours were discovered by palpation in 14 mice over an 18-month period.

3,4-*benzpyrene*

An initial inflammation similar to that of the control was seen up to 3 days after implantation. The amount of exudate had decreased by the 7th day. However, although fibroblasts were present scattered around the locality of the filter, a connective-tissue capsule had not formed. Macrophages and leucocytes were also still present throughout the tissue. Cells present near local blood vessels were proliferative. However, autoradiographic studies illustrated that very few of the cells within

FIG. 2.—Filter (MF) and surrounding tissues 8 weeks after the implantation of BP. The sub-pannicular connective tissue (SpC) contains few cells. The panniculus (PC) has degenerated and consists of single cells permeated by exudate. D, dermis. ×300.

the direct area of the filter had incorporated $^3$H-TdR. Four weeks after implantation, macrophages, leucocytes, fibroblasts and FBG cells were present in the exudative connective tissue surrounding the filter. Numbers of these cells enclosed crystal-shaped inclusions, presumably of BP. Very few cells near the filter surface were proliferative. Occasionally large fibroblast-like spindle cells were seen to incorporate $^3$H-TdR on the outskirts of the tissue reaction. The panniculus carnosus and deeper muscle tissue, especially when close to the filter, often degenerated. Cells within these areas were proliferative. The walls of blood vessels in the connective tissue adjacent to the filter were sometimes thickened and eosinophilic. The nuclei of the constituent cells were large, and autoradiography showed that many had incorporated $^3$H-TdR. Mammary ducts in close proximity to the filter were often hyperplastic. A very similar tissue response was noted 8 weeks after implantation (Fig. 2). The general nature of the tissue reaction remained unchanged until the formation of s.c. tumours. A number of proliferative events in the area of the filter could be identified during this period:

1. Cell foci within areas of degenerating pannicular and deeper muscle tissue occasionally showed a high rate of incorporation of $^3$H-TdR. This muscle tissue was usually separated from the filter by a layer of oedematous connective tissue, but was occasionally in direct contact with the filter surface. The electron microscope revealed muscle-like cells free in small or large groups in the fluid surrounding the filter. Fig. 3 depicts cells that had actin and myosin filaments of a regular structure; Z-bands, M-bands and H-bands were apparent. Nuclei were often irregular and mitochondria were numerous. Myelin bundles and whorls were seen. Bundles of fenestrated organelles composed of a system of tubules and cisternae were occasionally seen, other cells were large,

FIG. 3.—Aberrant muscle cells in the sub-pannicular connective tissue surrounding the implanted filter. Myelin whorls (MW) and fenestrated organelles (FO) were present. Ex, exudate. ×4,500.

FIG. 4a.—Autoradiograph of implant site tissue 9 weeks after treatment with BP. Large DNA-synthetic cells (SC) near the filter surface (MF) have incorporated thymidine. The cells in the surrounding oedematous sub-pannicular connective tissue are non-proliferative (NPC). ×300.

FIG. 4b.—Autoradiograph of proliferative focus of cells (PF) occurring on the filter surface (MF) 14 weeks after the implantation of BP. ST, subcutaneous tissues. ×300.

FIG. 5.—Cells of proliferative filter-attached focus 11 weeks after the implantation of BP. Note Golgi complexes (GC); dilated RER (ER); mitochondria containing smooth-membraned vesicles (Vm) and vesicles free in the cytoplasm (Vg). ×11,250.

strap-like and multinucleate, containing sheaves of filaments irregularly distributed in the cytoplasm. Z-band material was prominent. Nuclei were large and irregular. Only small amounts of endoplastic reticulum were apparent, but large numbers of ribosomes, usually grouped together as polyribosomes, were scattered throughout the cytoplasm. Many mitochondria were present. These cells were very similar to those seen in the final tumour (Fig. 8).

2. In contrast to the surrounding tissues, only the cells on or near the filter surface were seen to have incorporated $^{3}$H-TdR (Fig. 4*a*). These cells had very large oval or irregular nuclei with prominent nucleoli and many chromatin granules. Nuclear membranes had many invaginations. The cytoplasm was scanty with perinuclear basophilia.

3. Small foci of cells with similar morphology were seen on or near the filter surface (Fig. 4*b*). Cells were in intimate contact with each other, and only small amounts of collagen or reticulin were present. Up to 1 in 4 of these cells incorporated $^{3}$H-TdR. Some filters had several of such foci on their surface. Sections with similar but larger foci of cells were observed under the electron microscope. Cells showed the morphological features illustrated in Figs. 5 and 6. Cells tended to be elongated with a single nucleus. Nuclei were large and irregular in shape, with prominent areas of heterochromatin. The cytoplasm contained a prominent dilated rough endoplastic reticulum. Many Golgi complexes were present (Fig. 5). Mitochondria were numerous and often contained rounded smooth vesicles (Fig. 5). However, cytoplasmic structures of a similar size to mitochondria and bordered by a double membrane were frequently present, containing a dense array of these smooth-surfaced vesicles. These vesicles were also free in the cytoplasm of the cells (Figs. 5 & 6). Annulate lamellar structures (Fig. 6)

FIG. 6.—As Fig. 5. Note annulate lamellar structures (AL); microtubules (Mt); dilated RER (ER); smooth-surfaced vesicles (V); lysosomes (Ly); fibrillar material diffusely distributed and formed into sheaves (Mf) and extracellular amorphous material consistent with basal lamellae (AM). A= ×15,000. B= ×4,500.

FIG. 7a.—Type 1 cell tumour. ×300. b.—Type 2 cell tumour. ×300.

FIG. 8.—Cells from Type 2 cell tumour that contained bundles of myofilaments organized to form sarcomeres (S) with prominent Z-bands (Z). ×7,500.

were seen in these cells, consisting of regular stacks of membranes. Microfilaments were prominent. They were either distributed diffusely throughout the cytoplasm or condensed into bundles of sheaves adjacent to the plasma membrane (Fig. 6). Round electron-dense bodies consistent with lyososomes were present in many of the cells (Fig. 6). Pericellular deposits of amorphous or fibrillar substance resembling basal membranes were occasionally present.

Large s.c. tumours 1–1·5 cm in diameter formed in 17 mice, in a mean time of 105 days. These tumours were undifferentiated sarcomas consisting of either one or both of 2 major cell components (11 Type 1, 3 Type 2, and 3 mixed Type 1 and 2). Necrosis, inflammation and haemorrhage were present. Blood vessels were numerous and immature. All tumours invaded the surrounding tissues, including skin, muscle, fat and connective tissue.

*Type* 1

Large spindle-shaped cells of varying sizes (Fig. 7*a*) with large irregularly shaped nuclei. Nuclei were basophilic with a fine chromatin network and numerous chromatin granules. Cells usually contained one nucleus, although multinucleate cells were present with up to 20 or more nuclei. Multinucleate cells were rounded or strap-like. The cytoplasm of the cells was not extensive, and had a pronounced perinuclear basophilia. Cells formed swirling patterns of growth. Only very small quantities of reticulin or collagen were present. Ultrastructurally these cells ranged from those with the same morphological features as those illustrated in Figs. 5 and 6 and containing all the noted cytoplasmic inclusions, to those similar to that illustrated in Fig. 9*b*. These cells contained only small amounts of RER, many polyribosomes and little fibrillar material.

FIG. 9a.—Cell from Type 2 cell tumour that contained sheaves of filaments (Mf); polyribosomes (Pr); smooth-surfaced vacuoles (V) and numerous mitochondria (M). × 15,000. b.—Cell from Type 2 cell tumour that was of a similar nature to that illustrated in (a) but contained no bundles of filaments. × 15,000.

*Type* 2

The nuclei of these cells were of a similar size to those of the Type 1 cells (Fig. 7*b*). The nuclear matrix had a less prominent chromatin network. The cells had an abundant acidophilic cytoplasm.

Multinucleate cells were prominent and grew in interlacing straps and bundles. Cross-striations were noted in some of these cells. Only small amounts of collagen or reticulin were present. Many of the large multinucleate cells of these tumours were morphologically similar to the Type 1 multinucleate cells. As in the Type 1 tumours, the multinucleate cells also often contained an abundant acidophilic cytoplasm. Ultrastructurally, the constituent cells of the Type 2 tumours varied in morphology as shown in Figs. 8, 9*a* and 9*b*. Some of the cells (Fig. 8) were large and irregular. They contained filaments arranged to form sarcomeres with Z-bands. Mitochondria-free ribosomes and polysomes were present. Only small amounts of endoplastic reticulum were seen. Nuclei were usually of an irregular shape.

Other cells (Fig. 9*a*) contained smooth-surfaced vesicles scattered throughout the cytoplasm with irregularly distributed fibrillar material which was also present in the form of sheaves. Z-band material was not present.

A third type of cell (Fig. 9*b*) contained little endoplastic reticulum, many free ribosomes, polyribosomes, smooth-surfaced vesicles, and small amounts of randomly scattered fibrillar material. However, no sheaves of fibrils were present All tumours of Type 2 cells that were examined contained this variety of cells.

### DISCUSSION

In these experiments the requirement for a non-tumorigenic solid base for the implant was of primary importance. We have confirmed the findings of Goldhaber (1961) and Karp *et al.* (1973) that Millipore filters of pore size 0·22 $\mu$m are non-tumorigenic after s.c. implantation.

The incorporation of pyrene into the gelatin overlay did not substantially alter the tissue reaction to implantation. The material remaining in the subcutis became coated by a capsule similar to that seen with control implants, but somewhat thicker. No tumours were found.

The incorporation of 5 mg of BP into the gelatin overlay profoundly altered the course of the tissue response. An early feature of the tissue reaction was a prolonged exudation and infiltration by macrophages and lymphocytes. The mechanism of this is poorly understood, but various authors (Klimenko, 1958; Vasiliev *et al.*, 1962) have suggested that the lymphocytic infiltration may occur as a result of the local immune reaction to tissue proteins modified by the carcinogens. Indeed, Curtis *et al.* (1978) have shown that BP induces a pronounced immune response, and their findings suggested that this response is an important component of the carcinogenic process. Further, the tumour-inhibitory effect of specific cytotoxic lymphoid cells (Berczi & Sehon, 1977) may explain the predominance of lymphocytes in the BP-induced tissue reactions. In the present study the normal fibroblast response to implantation was inhibited. This particular aspect of carcinogen treatment has also been noted by Vasiliev *et al.* (1962), who demonstrated an inhibition of fibroblastic differentiation and growth around paraffin pellets containing carcinogens. Similar findings (Hooson *et al.*, 1971, 1973) followed repeated injections of water-soluble carcinogens into the subcutis. Moreover, carcinogenic hydrocarbons inhibit the proliferation of fibroblasts *in vitro*, whilst chemically related but non-carcinogenic hydrocarbons do not (Vasiliev & Guelstein, 1963). These observations are consistent with Evensen's view (1961) that carcinogens interfere with the synthesis of DNA and the mitotic process.

The large aberrant cells that were observed actively incorporating $^3$H-TdR on or near the filter surface about 8 weeks after implantation were in contrast to the surrounding non-proliferative tissue. These cells and those noted in the local muscle tissue were the most likely progenitors of the proliferative foci that ultimately arose adjacent to the implanted filter. The cells in these foci, both those consisting of aberrant spindle cells and those consisting of multinucleate muscle-like cells, together with the subsequent tumours, exhibited morphological characteristics similar to tumour cells described elsewhere. First, the spindle cells of the early foci and the differentiated Type 1 cell tumours were morphologically similar to tumour cells described by Johnson *et al.* (1973) that developed after the s.c. implantation of plastics. As with the present work, in addition to the usual subcellular organelles, prominent constituents of many of the cells were microfilaments either scattered diffusely throughout the cytoplasm or concentrated in the form of bundles or sheaves. However, the occurrence of microfilaments does not appear to incriminate a specific mesenchymal cell type as tumour originator (Johnson *et al.*, 1973). These structures have been described in a variety of mesenchymal tumours, including epithelioid sarcomas (Frable *et al.*, 1973), haemangiopericytomas (Murad & Von Haam, 1968), fibrosarcomas (Crocker & Murad, 1969) and lieomyoblastomas (Cornog, 1969). In addition to the cellular constituents noted in foreign-body sarcomas (Johnson *et al.*, 1973), our studies illustrated the presence of annulate lamellar structures which have been more frequently encountered in malignant cells than in normal cells (Elliot & Arkelger, 1966; Chambers & Weiser, 1964; Merkow *et al.*, 1967).

The fibroblast has commonly been implicated as the originator cell of chemically induced s.c. sarcomas (Vasiliev *et al.*, 1962; Carter, 1970) and the sub-cellular features of the Type 1 cell tumours reported here are consistent with this hypothesis. However, as with foreign-body-induced sarcomas (Johnson *et al.*, 1973; Brand *et al.*, 1976) another cell type such as the pericyte may be implicated as the originator of the spindle-cell tumours. The pericyte deserves special consideration as a possible cell of origin, in that it is a local pluripotential mesenchymal cell, and this could account for the subcellular variety within and between tumours. Also, it possesses subcellular features which are compatible with those seen in the tumour cells.

The proliferative foci of aberrant muscle-like cells noted in some of the sections were morphologically similar to rhabdomyosarcoma cells described by Friedman & Bird (1969). Variously distributed actin and myosin filaments were present as were Z-bands. Many of the cells had fenestrated organelles, consisting of a system of tubules and cisternae similar to those described by Hagopian & Spiro (1967). Myelin clusters were also present. These cells probably originated from the panniculus carnosus or deeper muscle tissue.

The Type 2 tumour cells noted here were also of very similar morphology to the rhabdomyosarcomas described by Friedman & Bird (1969), who showed that it was possible to discriminate between at least 4 types of such tumours, depending on their state of differentiation. In the present study the Type 2 tumour cells also varied in their state of differentiation, ranging from those with small numbers of cytoplasmic filaments of no fixed length often localized in the perinuclear zone, to those containing organized actin and myosin filaments arranged to form myofilaments with prominent Z-banding.

These studies indicate that the cellular progression of neoplasia can take 2 main routes following the s.c. implantation of BP. Firstly, the transformation of fibroblast or pericyte-like cells with their subsequent progression through filter-attached foci to sarcomas that vary in their state of differentiation. Secondly, the transformation of cells of the panniculus carnosus or deeper muscle, and their progression to sarcomas that also vary in their state of differentiation.

However, the morphological distinction between the undifferentiated Type 1 tumour cells, described here, and the undifferentiated Type 2 tumour cells was very restricted. Both types of cell had a limited dilated rough endoplasmic reticulum, many mitochondria and polyribosomes, and very small quantities of fibrillar material. Both were found to form multinucleate structures. Although the results of these investigations indicate this dual progression of neoplasia, the possibility cannot be ruled out that both histological groups of tumours arose, at least in some cases, from the same cell origin.

A number of factors indicate that a pluripotential cell such as the pericyte is probably capable of producing this variety of tumours. As already noted, Johnson *et al.* (1973) implicated the pericyte as a possible progenitor of foreign-body-induced sarcomas. Furthermore, Hard & Butler (1971) found that cells of tumours arising in the kidney after treatment of rats with dimethylnitrosamine displayed a broad histological spectrum, including cells similar to fibroblasts, pericytes, smooth-muscle cells and rhabdomyoblasts. Despite this there was no basis for a type subdivision. It was suggested that the pericyte was the most likely progenitor cell. Thus the pericyte is probably capable of yielding tumours consistent with both cell types noted in these studies. This hypothesis is at present being examined in our laboratories by the use of *in vivo/in vitro* tissue-culture techniques to study the early stages of tumorigenesis by BP in the subcutis.

The authors are grateful to Mrs E. Penny for skilled technical assistance in the preparation of electron-microscope samples used in the study, and to colleagues at CTL, particularly Dr I. Pratt, for their constructive criticism during the course of the investigation.

### REFERENCES

BERCZI, I. & SEHON, A. H. (1977) Tumour inhibition by effector cells cultured from progressing sarcomas. *Immunol. Commun.*, **6**, 617.

BRAND, K. G., JOHNSON, K. H. & BUOEN, L. C. (1976) Foreign-body tumourigenesis. *Crit. Rev. Toxicol.*, p. 353.

CARTER, R. L. (1969) Early development of injection site sarcomas in rats: a study of tumours induced by iron dextrans. *Br. J. Cancer*, **23**, 559.

CARTER, R. L. (1970) Induced subcutaneous sarcomata: Their development and critical appraisal. In *Metabolic Aspects of Food Safety*. Ed. J. C. Roe. Oxford: Blackwell.

CHAMBERS, V. C. & WEISER, R. S. (1964) Annulate lamellae in Sarcoma 1 cells. *J. Cell Biol.*, **21**, 133.

CORNOG, J. L. (1969) The ultrastructure of leiomyoblastomas with comments on the light microscope morphology. *Arch. Pathol.*, **87**, 404.

CROCKER, D. J. & MURAD, T. M. (1969) Ultrastructure of fibrosarcoma in a male breast. *Cancer*, **23**, 891.

CURTIS, G. L., RYAN, W. L. & STENBACK, F. (1978) Antibody stimulation of benzo[a]pyrene carcinogenesis. *Cancer Lett.*, **4**, 223.

ELLIOT, R. L. & ARKELGER, R. B. (1966) Fine structure of parathyroid adenomas with special reference to annulate lamellae and septate desmosomes. *Arch. Pathol.*, **81**, 200.

EVENSEN, A. (1961) Changes in the synthesis of deoxyribonucleic acid (DNA) and in mitotic count in epidermis of hairless mice after a single application of one per cent 3-methylcholanthrene in benzene. A preliminary report. *Acta Pathol. Microbiol. Scand. Suppl*, **148**, 43.

FRABLE, W. J., KAY, S., LAWRENCE, W. & SCHATZKI, P. F. (1973) Epithelioidsarcoma, an electron microscope study *Arch. Pathol.*, **95**, 8.

FRIEDMAN, I. & BIRD, E. S. (1969) Electron microscope investigation of experimental rhabdomyosarcoma. *J. Path.*, **97**, 375.

GOLDHABER, P. (1961) The influence of pore size on carcinogenesis of subcutaneously implanted Millipore filters. *Proc. Am. Assoc. Cancer Res.*, **3**, 228.

GRASSO, P. & GOLBERG, L. (1966) Early changes at the site of repeated subcutaneous injection of food colourings. *Fd. Cosmet. Toxicol.*, **4**, 269.

HAGOPIAN, M. & SPIRO, D. (1967) The sarcoplasmic reticulum and its association with the T system in an insect. *J. Cell Biol.*, **32**, 535.

HARD, G. C. & BUTLER, W. H. (1971) Ultrastructural analysis of renal mesenchymal tumours induced in the rat by dimethylnitrosamine. *Cancer Res.*, **31**, 348.

HOOSON, J., GRASSO, P. & GANGOLLI, S. D. (1971) Early reactions of the subcutaneous tissue to repeated injection of carcinogens in aqueous solutions. *Br. J. Cancer*, **25**, 505.

HOOSON, J., GRASSO, P. and GANGOLLI, S. D. (1973) Injection site tumours and preceding pathological changes in rats treated subcutaneously with surfactants and carcinogens. *Br. J. Cancer*, **27**, 230.

JOHNSON, K. H., GHOBRIAL, H. K. G., BOUEN, L. C., BRAND, I. & BRAND, K. C. (1973) Non-fibroblastic origin of foreign body sarcomas implicated by histological and electron microscopic studies. *Cancer Res.*, **33**, 3139.

KARP, R. D., JOHNSON, K. H., BOUEN, L. C., GHOBRIAL, H. K. G., BRAND, I. & BRAND, K. C. (1973) Tumourigenesis of Millipore filters in mice; ultrastructure of tissue reactions related to pore size. *J. Natl Cancer Inst.*, **51**, 1275.

KLIMENKO, E. D. (1958) Effect of the method of introduction and the dose of the carcinogenic stimulant on the development of morphological changes in the focus of tumour formation. *Bull. Exp. Biol. Med.*, **45**, 514.

LAVELLE, S. M. (1973) Action of histone on neoplastic and normal growth. *Int. J. Med. Science*, **142**, 58.

MERKOW, L. P., EPSTEIN, S. M., CAITO, B. J. & BARTUS, B. (1967) The cellular analysis of liver carcinogenesis; ultrastructural alterations and hyperplastic liver nodules induced by 2-fluorenylacetamide. *Cancer Res.*, **27**, 1712.

MURAD, T. M. & VON HAAM, E. (1968) Ultrastructure of myoepithelial cells in human mammary gland tumours. *Cancer*, **21**, 1137.

PURCHASE, I. F. H., LONGSTAFF, E., ASHBY, J., STYLES, J. A., ANDERSON, D., LEFEVRE, P. A. & WESTWOOD, F. R. (1976) Evaluation of six short-term tests for detecting organic chemical carcinogens and recommendations for their use. *Nature*, **264**, 624.

PURCHASE, I. F. H., LONGSTAFF, E., ASHBY, J. & 4 others (1978) An evaluation of six short-term tests for detecting organic chemical carcinogens. *Br. J. Cancer*, **37**, 873.

VASILIEV, J. M. (1959) Early changes in the subcutaneous connective tissue in rats after implantation of pellets containing carcinogenic polycyclic hydrocarbons. *J. Natl Cancer Inst.*, **23**, 441.

VASILIEV, J. M. & GUELSTEIN, V. I. (1963) Sensitivity of normal and neoplastic cells to the damaging action of carcinogenic substances: A review. *J. Natl Cancer Inst.*, **31**, 1123.

VASILIEV, J. M., OLSHEVSKAJA, L. V., RAIKHLIN, N. T. & IVANOVA, O. J. (1962) Comparative study of the alterations induced by 7,12-dimethylbenz[a]anthracene and polymer films in the subcutaneous connective tissue of rats. *J. Natl Cancer Inst.*, **28**, 515.


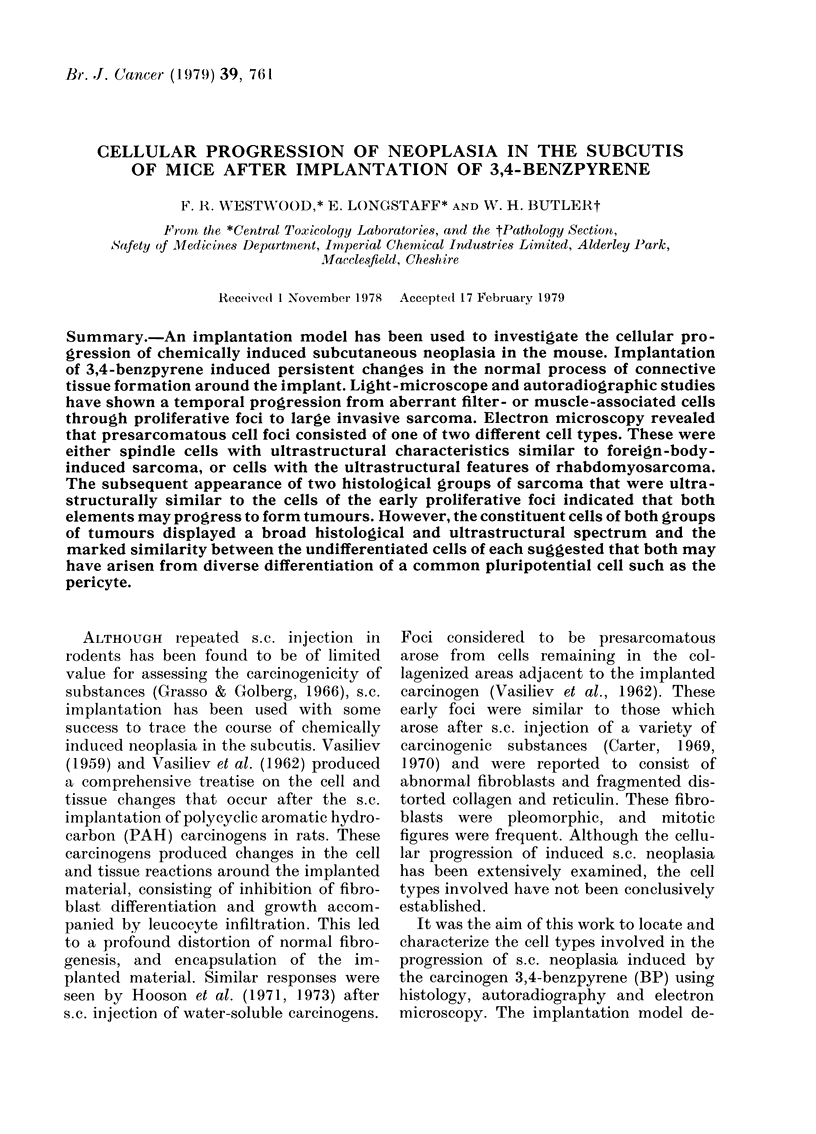

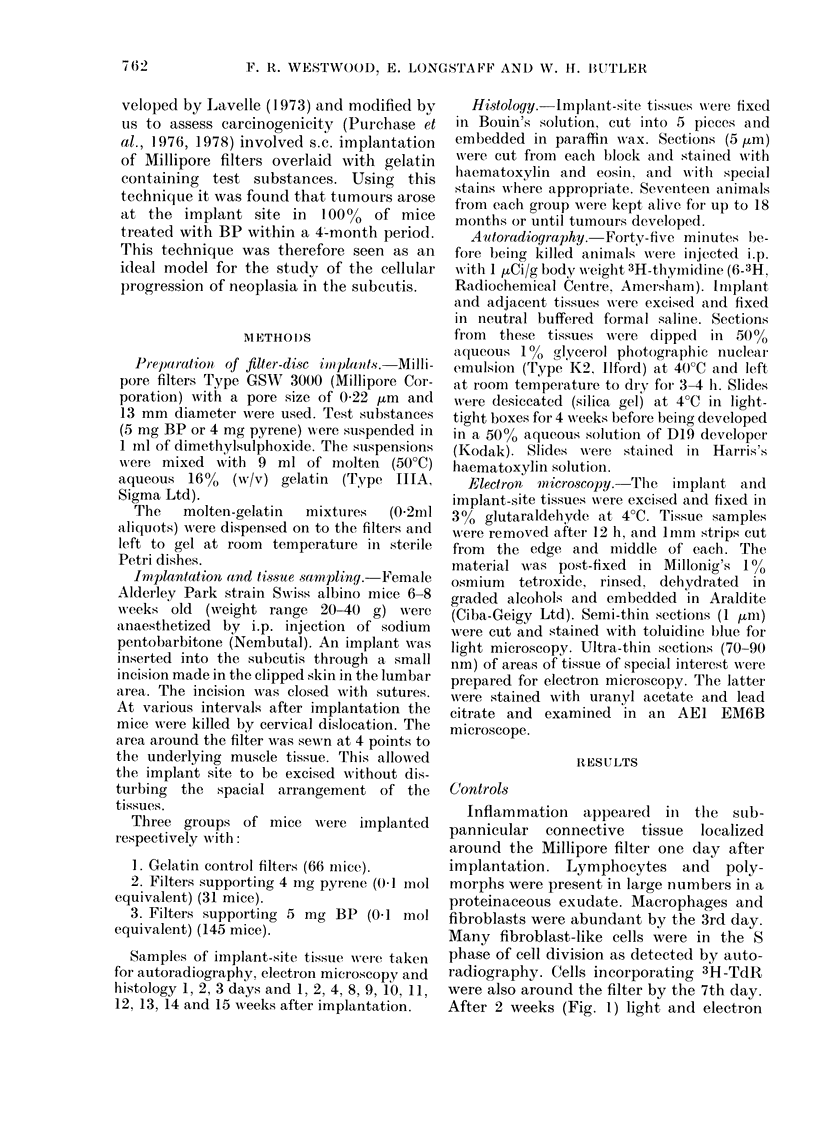

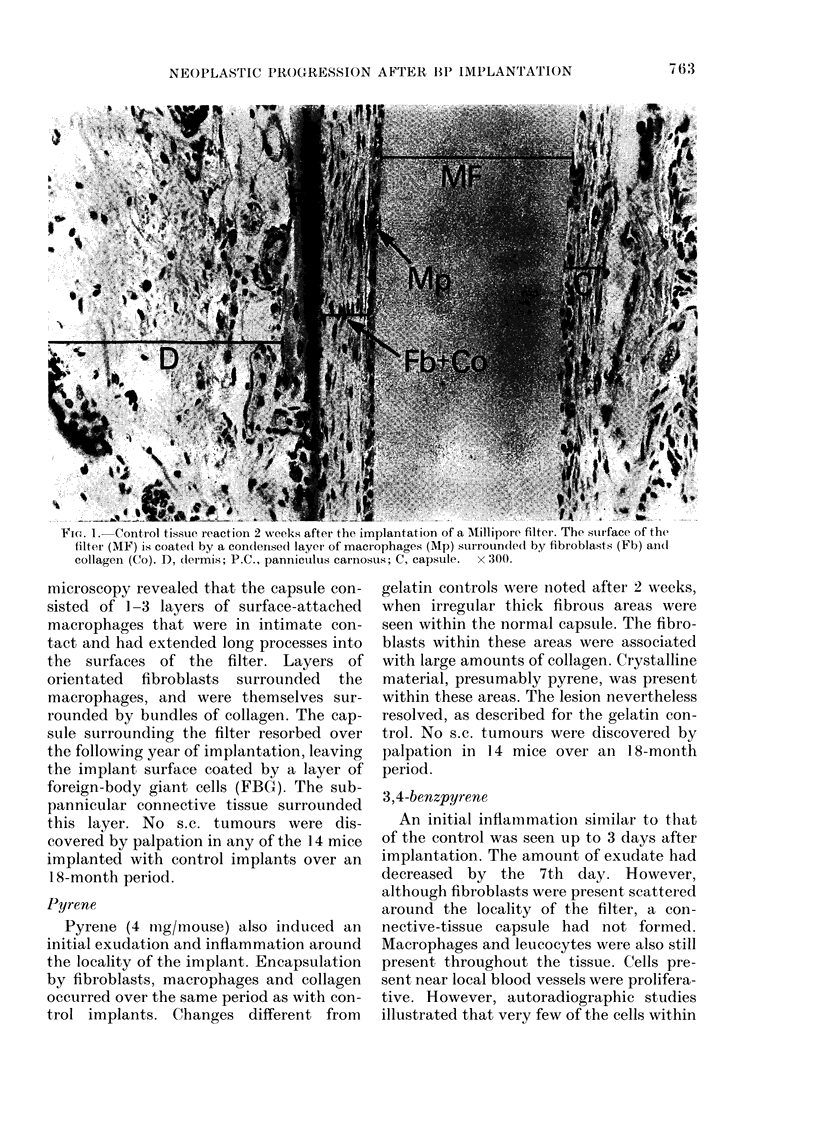

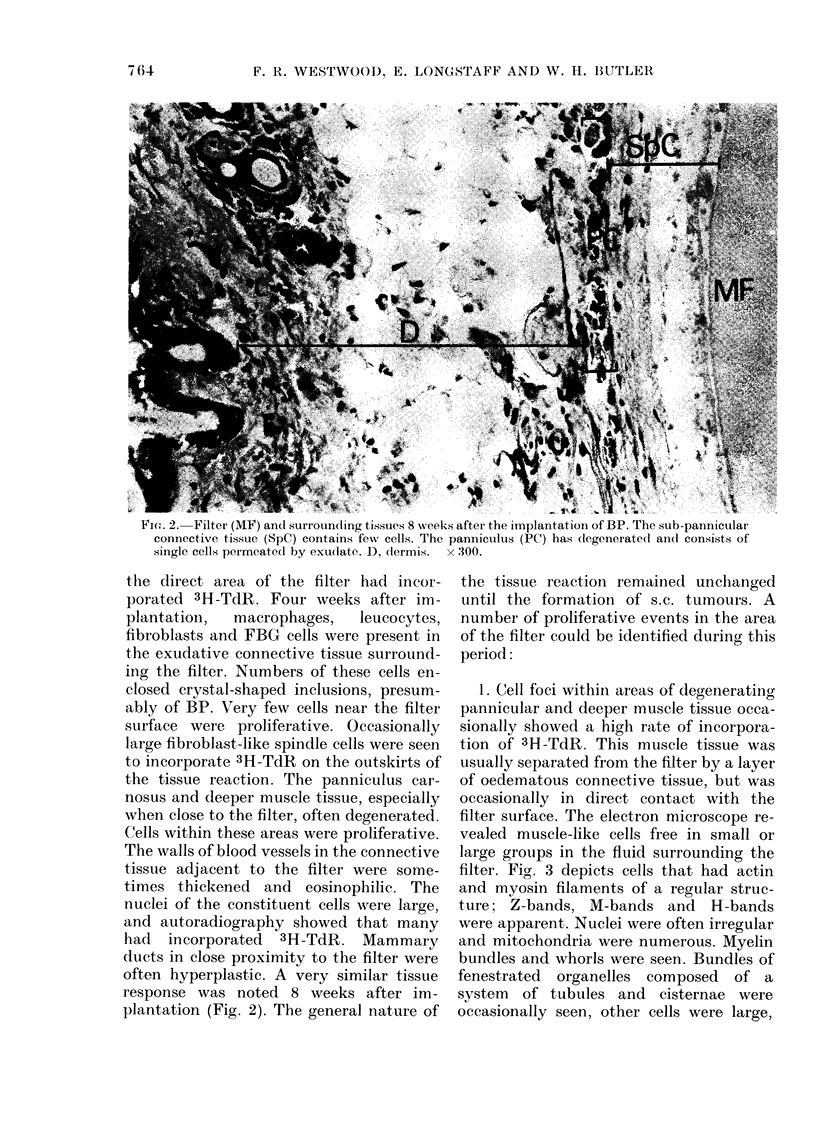

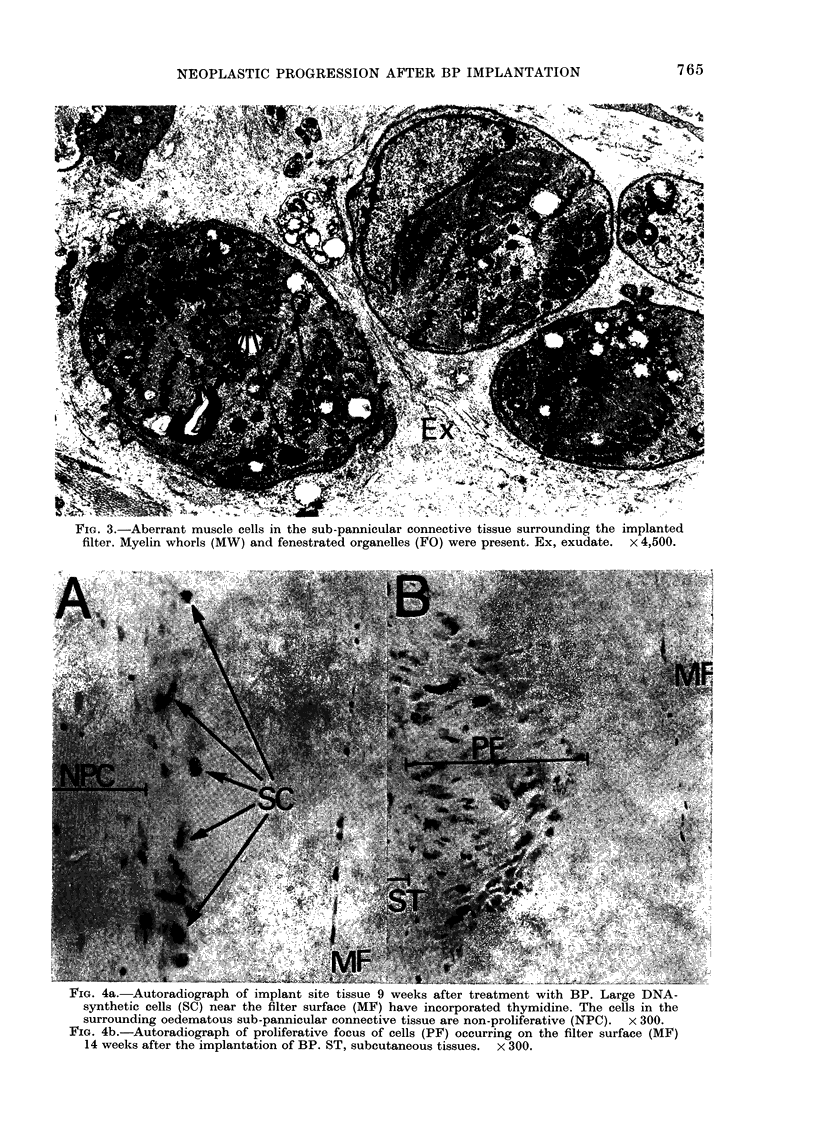

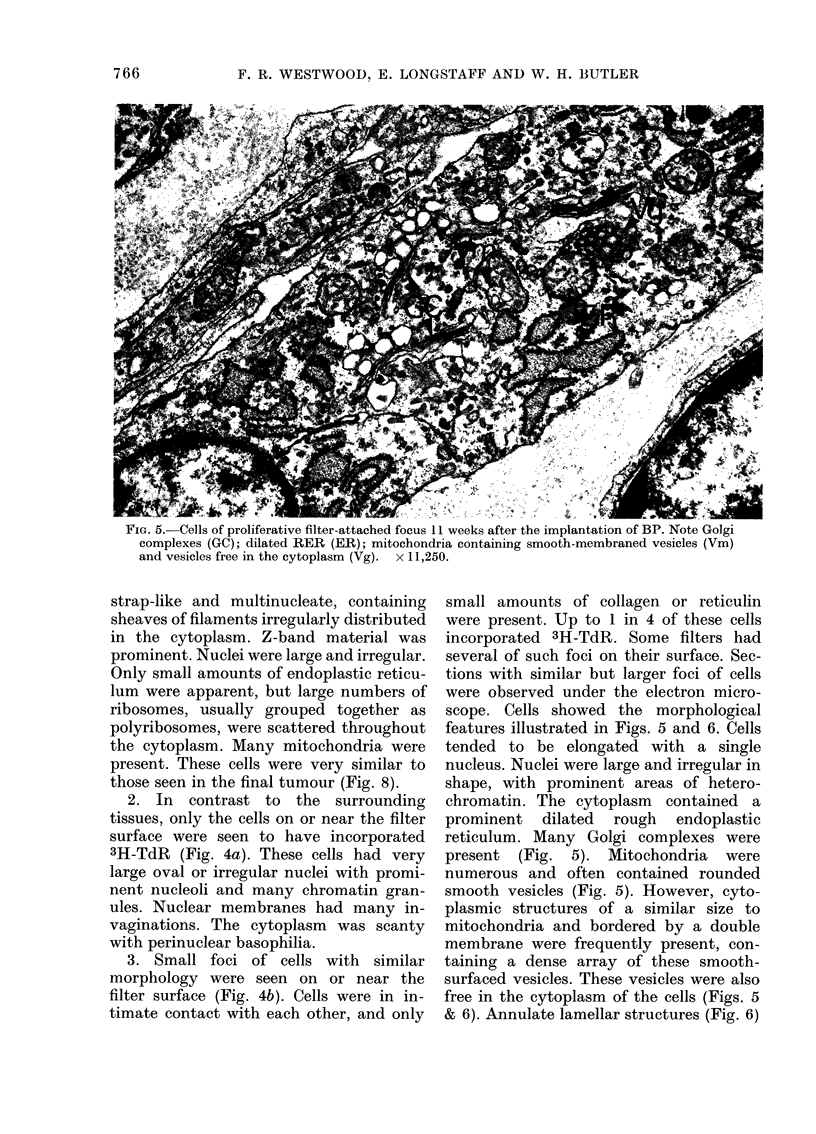

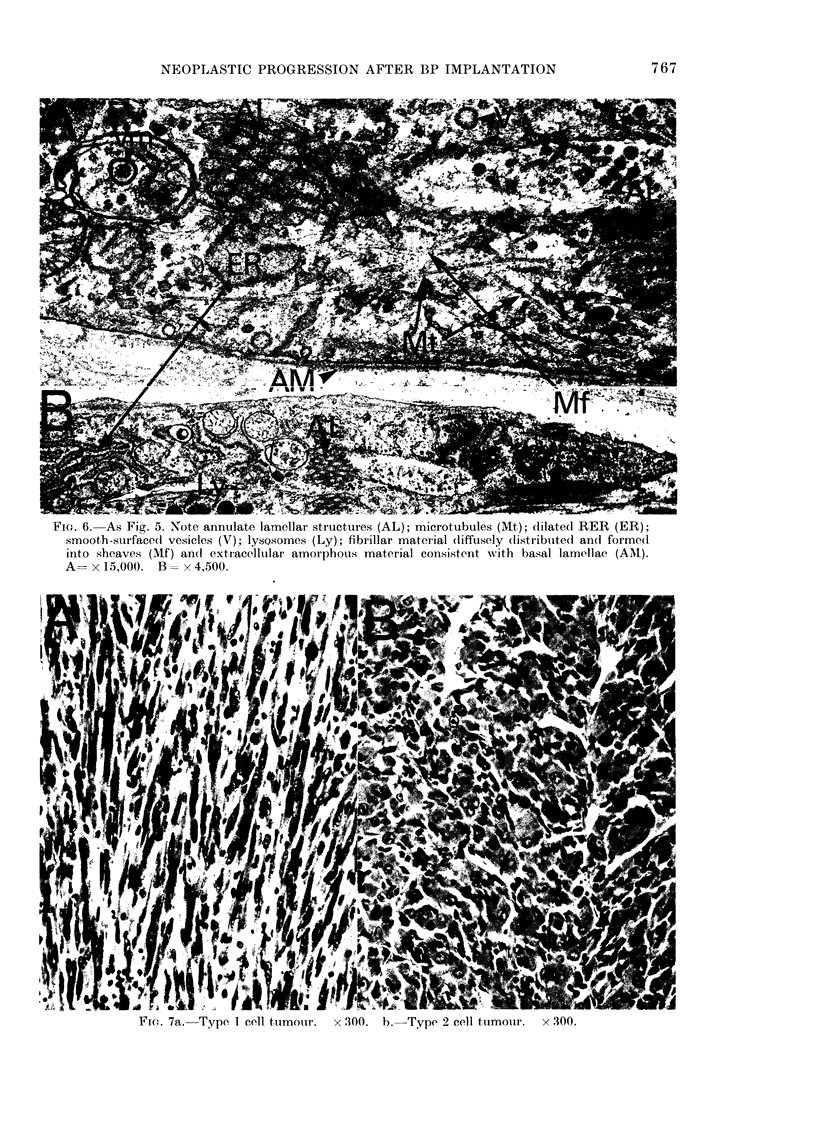

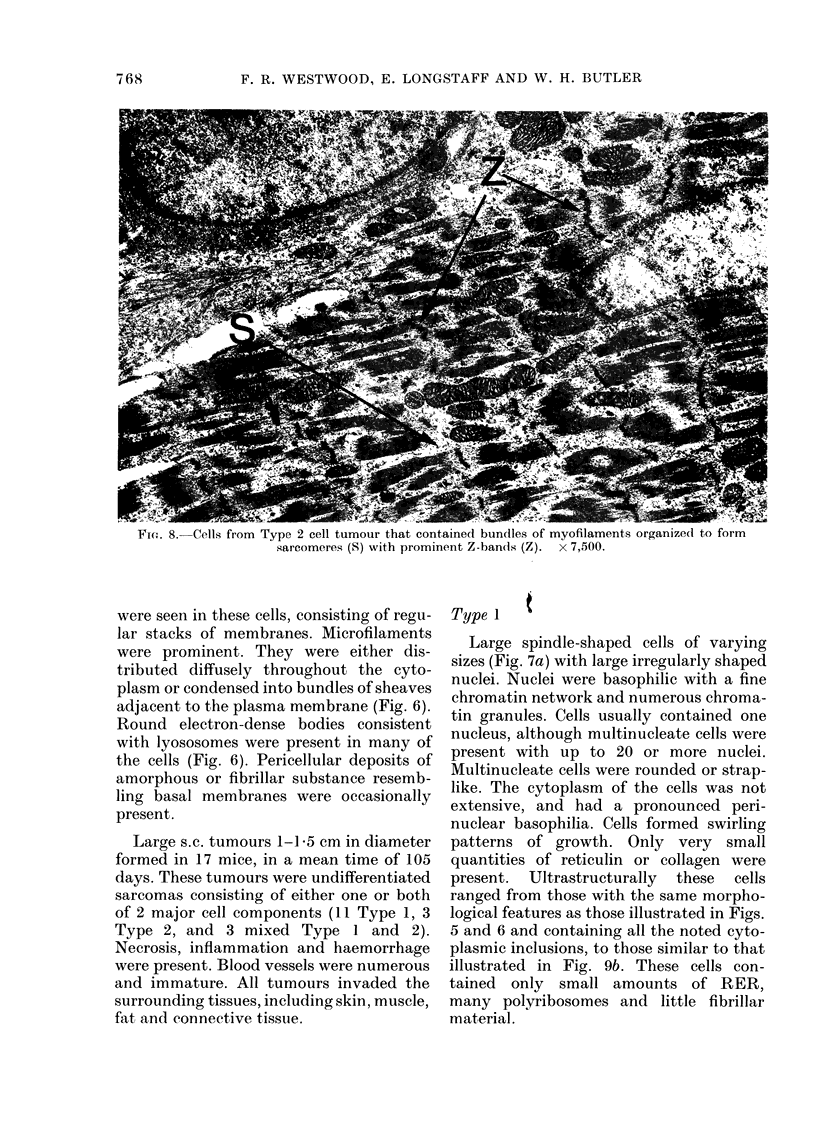

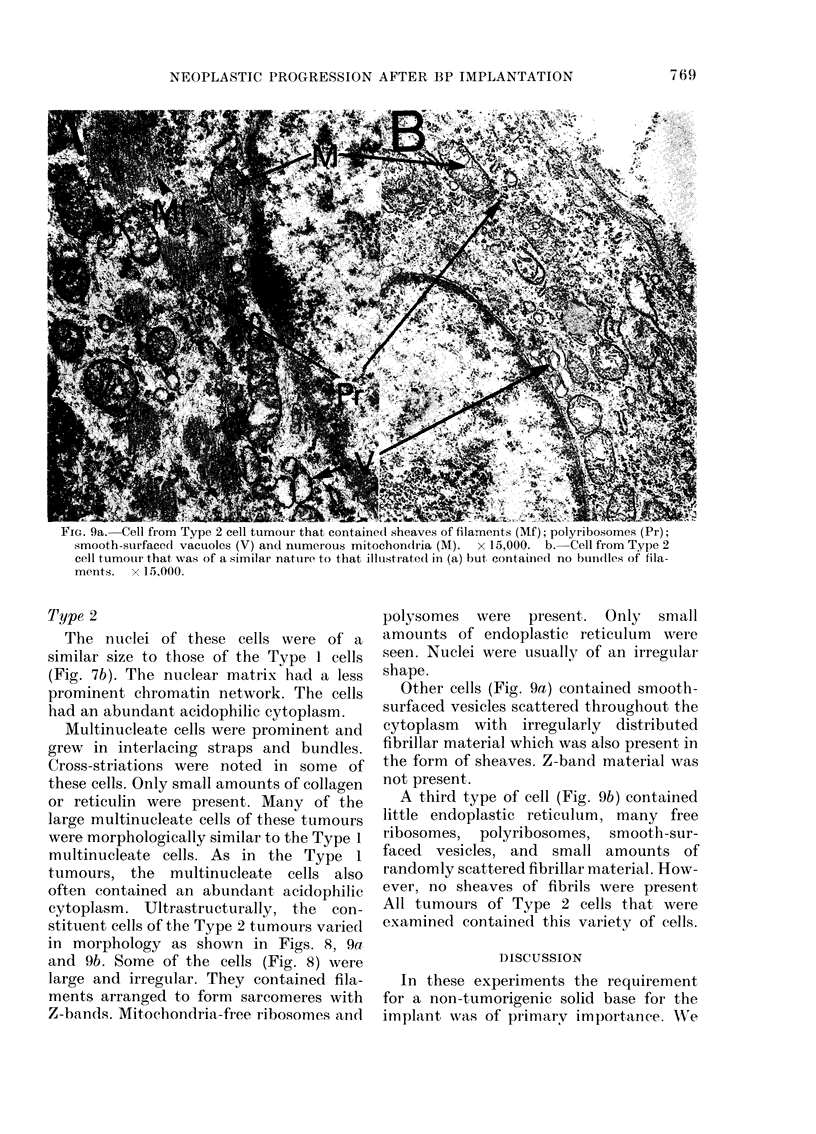

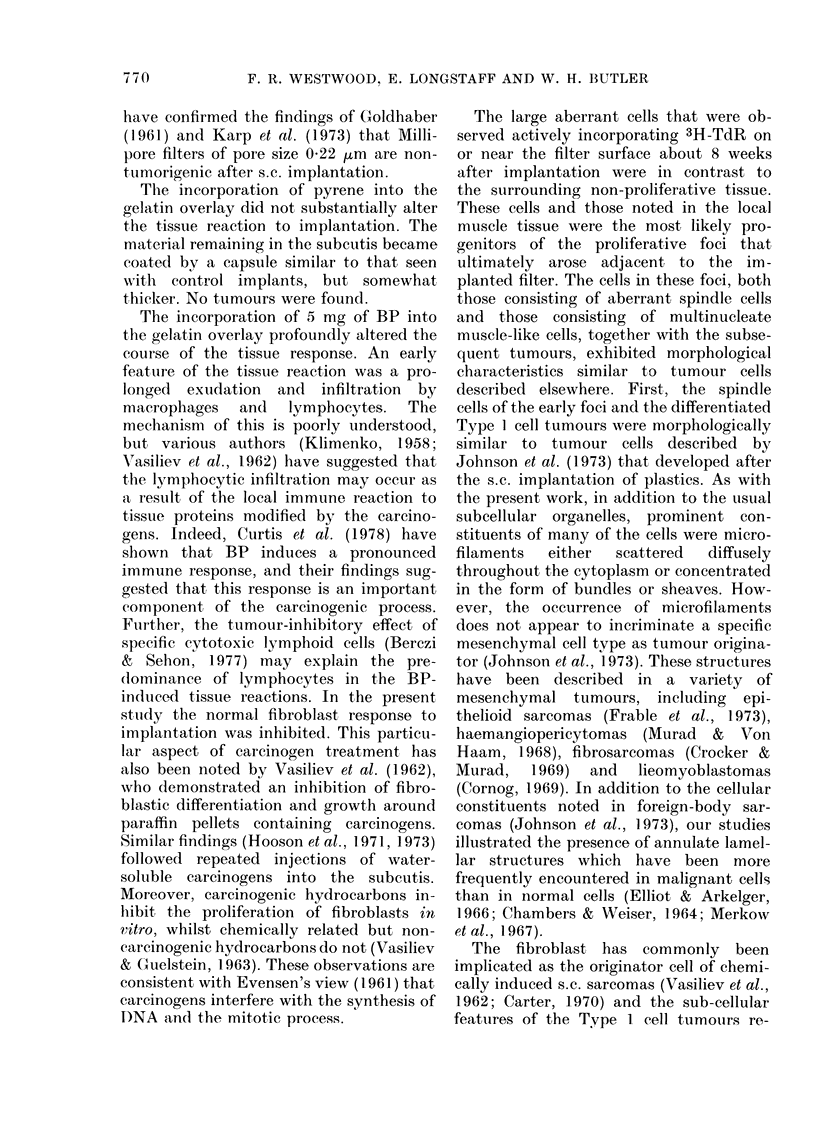

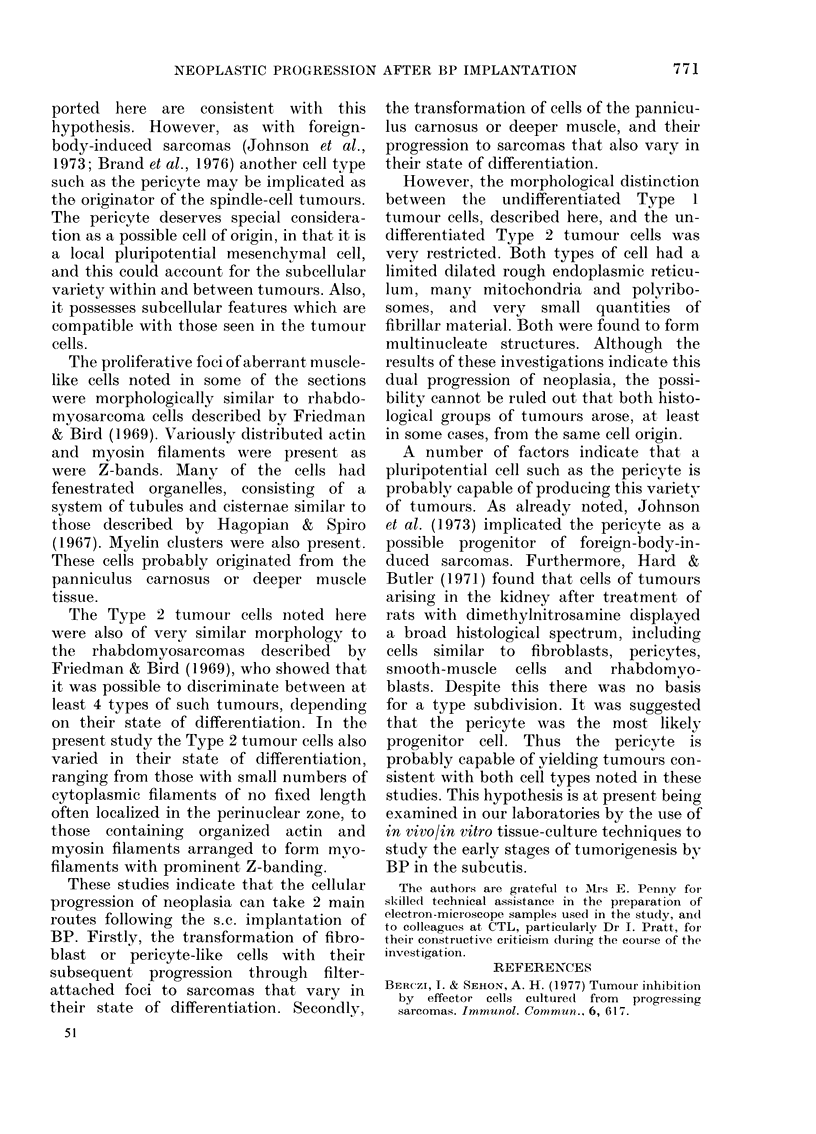

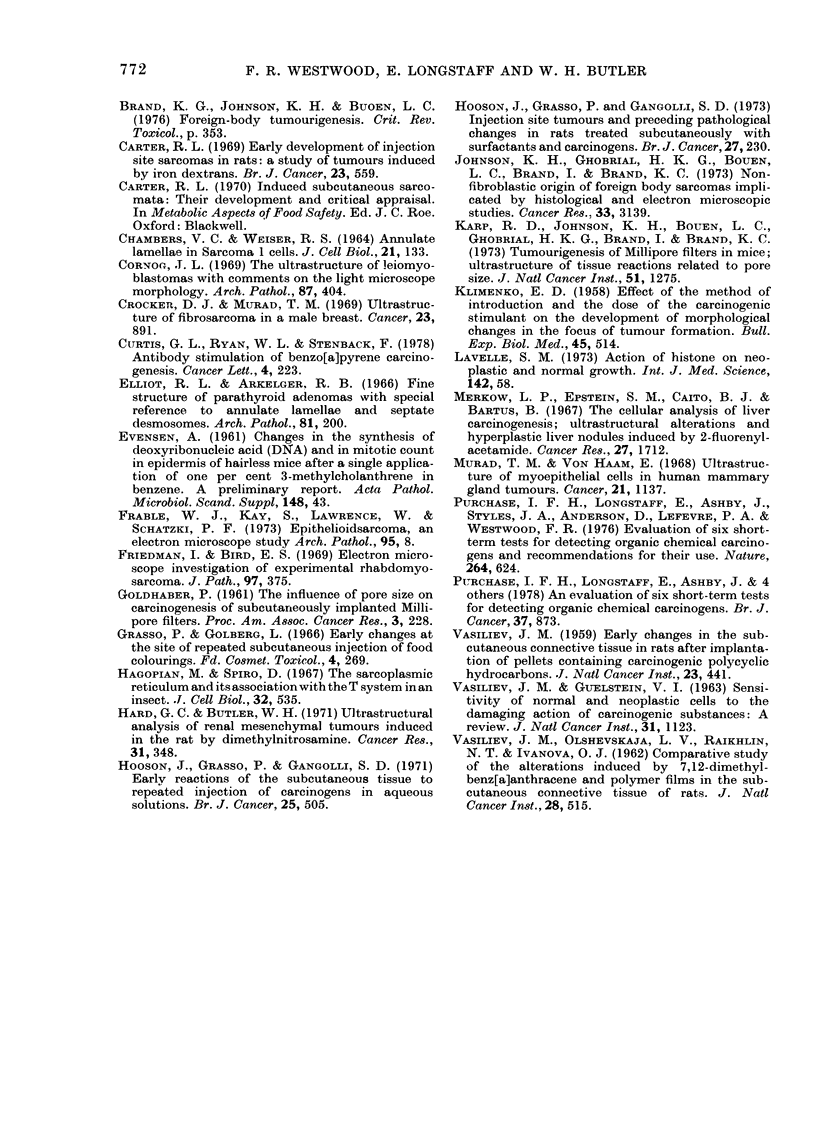


## References

[OCR_00763] Berczi I., Sehon A. H. (1977). Tumor inhibition by effector cells cultured from progressing sarcomas.. Immunol Commun.

[OCR_00772] Brand K. G., Johnson K. H., Buoen L. C. (1976). Foreign body tumorigenesis.. CRC Crit Rev Toxicol.

[OCR_00788] CHAMBERS V. C., WEISER R. S. (1964). ANNULATE LAMELLAE IN SARCOMA I CELLS.. J Cell Biol.

[OCR_00777] Carter R. L. (1969). Early development of injection-site sarcomas in rats: a study of tumours induced by iron-dextran.. Br J Cancer.

[OCR_00792] Cornog J. L. (1969). The ultrastructure of leiomyoblastoma. With comments on the light microscopic morphology.. Arch Pathol.

[OCR_00797] Crocker D. J., Murad T. M. (1969). Ultrastructure of fibrosarcoma in a male breast.. Cancer.

[OCR_00802] Curtis G. L., Ryan W. L., Stenbäck F. (1978). Antibody stimulation of benzo(a)pyrene carcinogenesis.. Cancer Lett.

[OCR_00813] EVENSEN A. (1961). Changes in the synthesis of deoxyribonucleic acid (DNA) and in mitotic count in epidermis of hairless mice after a single application of one per cent 3:methyl-cholanthrene in benzene. A preliminary report.. Acta Pathol Microbiol Scand Suppl.

[OCR_00821] Frable W. J., Kay S., Lawrence W., Schatzki P. F. (1973). Epithelioid sarcoma. An electron microscopic study.. Arch Pathol.

[OCR_00826] Friedmann I., Bird E. S. (1969). Electron-microscope investigation of experimental rhabdomyosarcoma.. J Pathol.

[OCR_00835] Grasso P., Golberg L. (1966). Early changes at the site of repeated subcutaneous injection of food colourings.. Food Cosmet Toxicol.

[OCR_00840] Hagopian M., Spiro D. (1967). The sarcoplasmic reticulum and its association with the T system in an insect.. J Cell Biol.

[OCR_00845] Hard G. C., Butler W. H. (1971). Ultrastructural analysis of renal mesenchymal tumor induced in the rat by dimethylnitrosamine.. Cancer Res.

[OCR_00851] Hooson J., Grasso P., Gangolli S. D. (1971). Early reactions of the subcutaneous tissue to repeated injections of carcinogens in aqueous solutions.. Br J Cancer.

[OCR_00857] Hooson J., Grasso P., Gangolli S. D. (1973). Injection site tumours and preceding pathological changes in rats treated subcutaneously with surfactants and carcinogens.. Br J Cancer.

[OCR_00862] Johnson K. H., Ghobrial H. K., Buoen L. C., Brand I., Brand K. G. (1973). Nonfibroblastic origin of foreign body sarcomas implicated by histological and electron microscopic studies.. Cancer Res.

[OCR_00869] Karp R. D., Johnson K. H., Buoen L. C., Ghobrial H. K., Brand I., Brand K. G. (1973). Tumorigenesis by Millipore filters in mice: histology and ultrastructure of tissue reactions as related to pore size.. J Natl Cancer Inst.

[OCR_00883] Lavelle S. M. (1973). Action of histone on neoplastic and normal growth.. Ir J Med Sci.

[OCR_00888] Merkow L. P., Epstein S. M., Caito B. J., Bartus B. (1967). The cellular analysis of liver carcinogenesis: ultrastructural alterations within hyperplastic liver nodules induced by 2-fluorenylacetamide.. Cancer Res.

[OCR_00895] Murad T. M., Von Haam E. (1968). Ultrastructure of myoepithelial cells in human mammary gland tumors.. Cancer.

[OCR_00908] Purchase I. F., Longstaff E., Ashby J., Styles J. A., Anderson D., Lefevre P. A., Westwood F. R. (1978). An evaluation of 6 short-term tests for detecting organic chemical carcinogens.. Br J Cancer.

[OCR_00900] Purchase I. F., Longstaff E., Ashby J., Styles J. A., Anderson D., Lefevre P. A., Westwood F. R. (1976). Evaluation of six short term tests for detecting organic chemical carcinogens and recommendations for their use.. Nature.

[OCR_00914] VASILIEV J. M. (1959). Early changes in the subcutaneous connective tissue of rats after implantation of pellets containing carcinogenic polycyclic hydrocarbons.. J Natl Cancer Inst.

[OCR_00920] VASILIEV J. M., GUELSTEIN V. I. (1963). SENSITIVITY OF NORMAL AND NEOPLASTIC CELLS TO THE DAMAGING ACTION OF CARCINOGENIC SUBSTANCES: A REVIEW.. J Natl Cancer Inst.

[OCR_00926] VASILIEV J. M., OLSHEVSKAJA L. V., RAIKHLIN N. T., IVANOVA O. J. (1962). Comparative study of alterations induced by 7,12-dimethylbenz[a]anthracene and polymer films in the subcutaneous connective tissue of rats.. J Natl Cancer Inst.

